# Acute Intoxication With Poison Hemlock (Conium maculatum)

**DOI:** 10.7759/cureus.80763

**Published:** 2025-03-18

**Authors:** João Nunes, Sergio Pina, Ana Carolina Oliveira, Javier Moreno, Pilar Pérez

**Affiliations:** 1 Critical Care Medicine, Unidade Local de Saúde (ULS) Algarve - Hospital de Faro, Faro, PRT

**Keywords:** acute intoxication, generalized tonic-clonic seizures, native plants, plant alkaloids, poison hemlock, poison ingestion

## Abstract

Poison hemlock (*Conium maculatum*) is regarded as one of the most poisonous plants worldwide, and it can easily be misidentified as edible species as celery, parsnip (for their leaves), or carrots (for their roots). Toxicity comes from piperidine alkaloids, which mimic nicotine effect on the autonomous nervous system giving a clinical picture of nicotinic syndrome with an initial excitatory phase and secondary inhibitory one. In Portugal, this plant is used as an ancient technique for river fishing. Death comes from respiratory arrest and hemodynamic collapse; rapid diagnosis and medical support are essential for successful treatment. We present two cases of different degrees of intoxication with total recovery.

## Introduction

In our region, intoxication emergencies are common. The Algarve (Portugal) is a very touristic location but also rural and agricultural one. Poison Hemlock is ubiquitous in this southern part of the country and readily accessible. Intoxications are somewhat always a stressful situation for clinicians and healthcare workers, even more with uncommon toxins.

## Case presentation

Two patients in their 30s, with no significant past medical history, were admitted to our hospital five hours after ingesting *Conium maculatum* with different degrees of severity of intoxication.

The female, who had the most severe intoxication, made a soup with the roots of this plant cultivated by themselves in their garden and they ate it for recreational purposes. Within three hours of ingestion, she developed tonic-clonic seizures with sphincter incontinence with one hour of duration that were refractory to 10 mg of diazepam and 15 mg of midazolam and was only stopped by the anesthetic propofol (40 mg). Afterward, she developed nausea and biliary vomiting with a post critical period and a Glasgow Coma Scale (GCS) of 8. She was evacuated by helicopter; upon arrival at the emergency department, she maintained a GCS of 8, with central but sluggish pupils, and initial vitals were a temperature of 36.7 °C, a blood pressure of 132/86 mmHg, and a respiratory rate of 17 breaths/minute. The physical examination, aside from the neurologic deficit, was normal. The laboratory results are shown in Table 1.

**Table 1:** Blood results

Test	Result	Reference range
Leukocytes	37,700/mm³	4,000-11,000/mm³
Hemoglobin	9.2 g/dL	13.8-17.2 g/dL (men) / 12.1-15.1 g/dL (women)
Platelets	430,000/mm³	150,000-450,000/mm³
INR (International Normalized Ratio)	1.0	0.8-1.1
AST (aspartate aminotransferase)	63 IU/L	0-40 IU/L
ALT (alanine aminotransferase)	24 IU/L	0-40 IU/L
Creatinine	1.1 mg/dL	0.6-1.2 mg/dL
Blood urea nitrogen (BUN)	0.6-1.2 mg/dL	7-20 mg/dL
Creatine phosphokinase (CPK)	3485 mcg/L	38-174 mcg/L

All serum electrolytes levels were normal. Creatine phosphokinase (CPK) was elevated at 3485 mcg/L. All other toxicology tests were normal. Her electrocardiogram showed normal sinus rhythm. She was admitted to the intensive care unit without experiencing any further seizures. The creatine phosphokinase level rose to 33,344 mcg/L, which was managed with intravenous fluids to maintain a diuresis of 200 cc/h, resulting in the normalization of all parameters. The exact cause of the rhabdomyolysis remains uncertain, but it may have been related to the seizures, intoxication, or other factors such as fever. Her neurological state required the most time to recover, with a period of delirium. She regained a GCS of 15 by the fourth day of her ICU stay. She was discharged well at the eighth day of hospitalization.

The male patient experienced a tonic-clonic seizure following ingestion of a single piece of spotted hemlock, with a duration of less than 10 minutes. Upon arrival, he was in a post-critical state with some slowness but maintained a GCS of 13. His laboratory results showed a mild elevation in CPK and severe hypophosphatemia, with phosphorus levels of 1.1 mg/dL (normal range 2.5-4.5 mg/dL), both of which were corrected with intravenous fluids. He regained his baseline neurological status within 48 hours and was discharged on the fifth day of hospitalization. He was managed in our intermediate care unit.

## Discussion

Poison hemlock (*Conium maculatum*) is one of the most poisonous plants known. It is a very common nitrophile weed species, belonging to the Apiaceae (formerly Umbelliferae) family, the carrot family. The juice or the extract of *C. maculatum* was the lethal poison which the Greek philosopher Socrates was condemned to drink (399 BC) and whose symptoms were brilliantly described by Plato, his best-known disciple [1].

Botanical characterization

**Figure 1:**
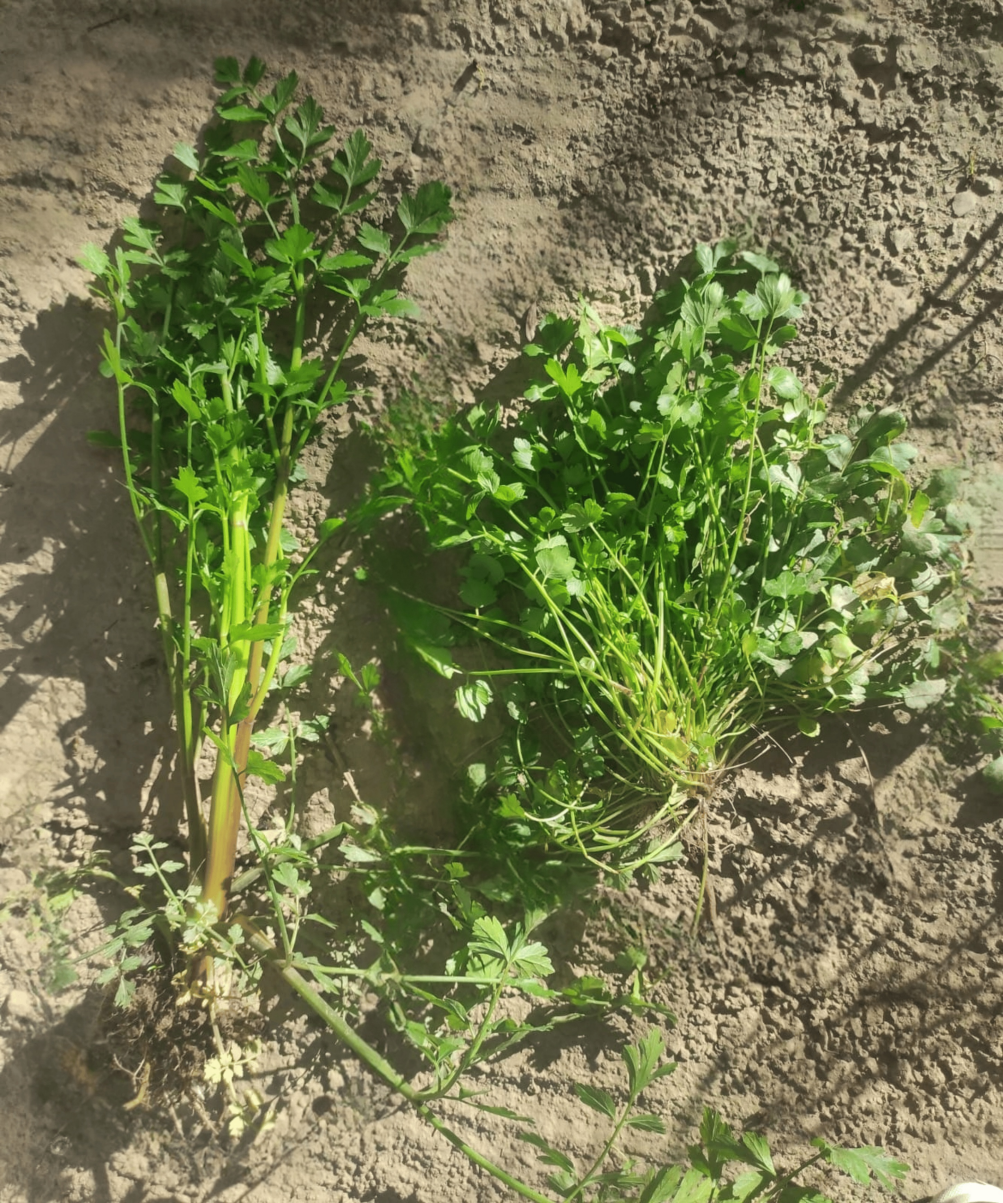
Poison hemlock (left) in comparison to non-poisonous coriander leaves (right).

**Figure 2:**
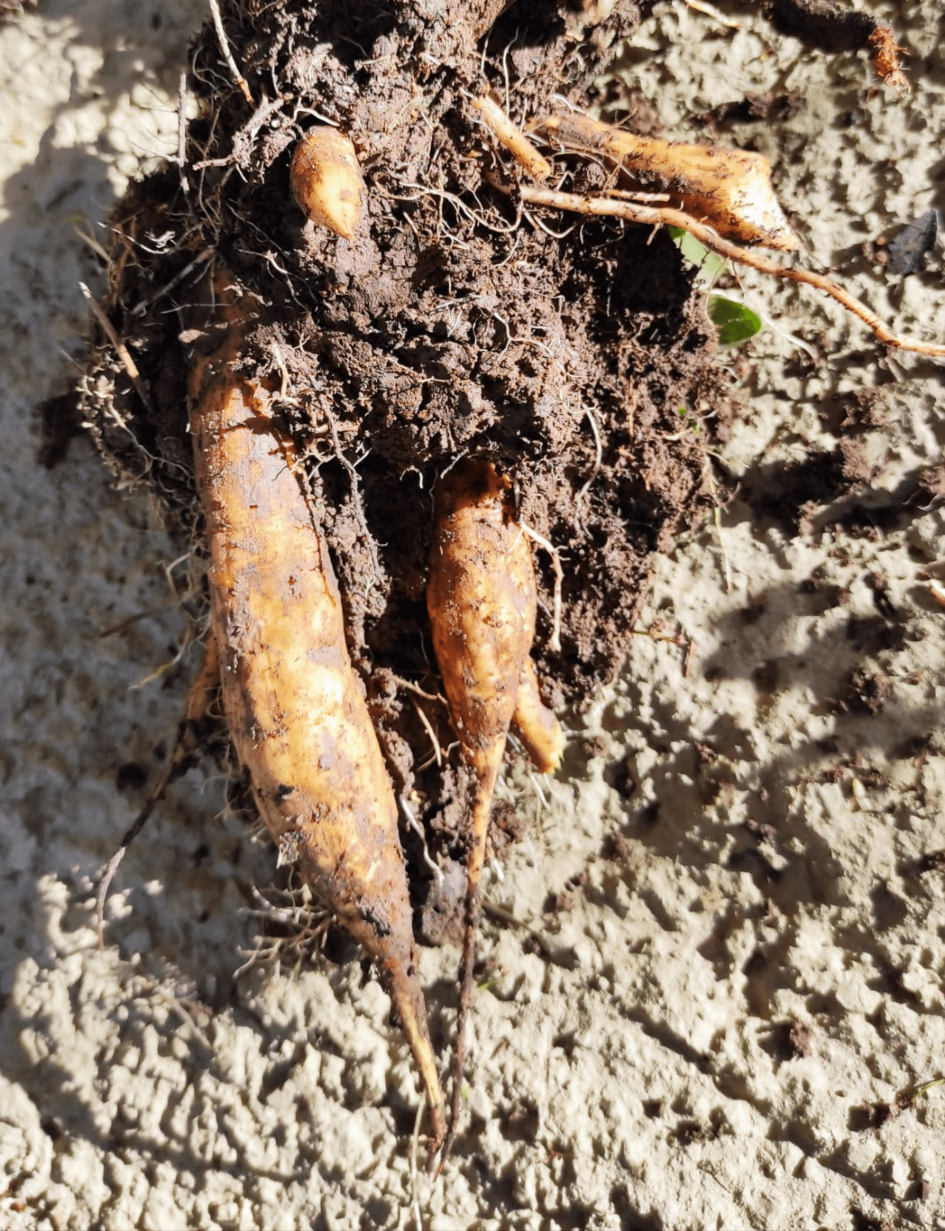
Poison hemlock root.

**Figure 3:**
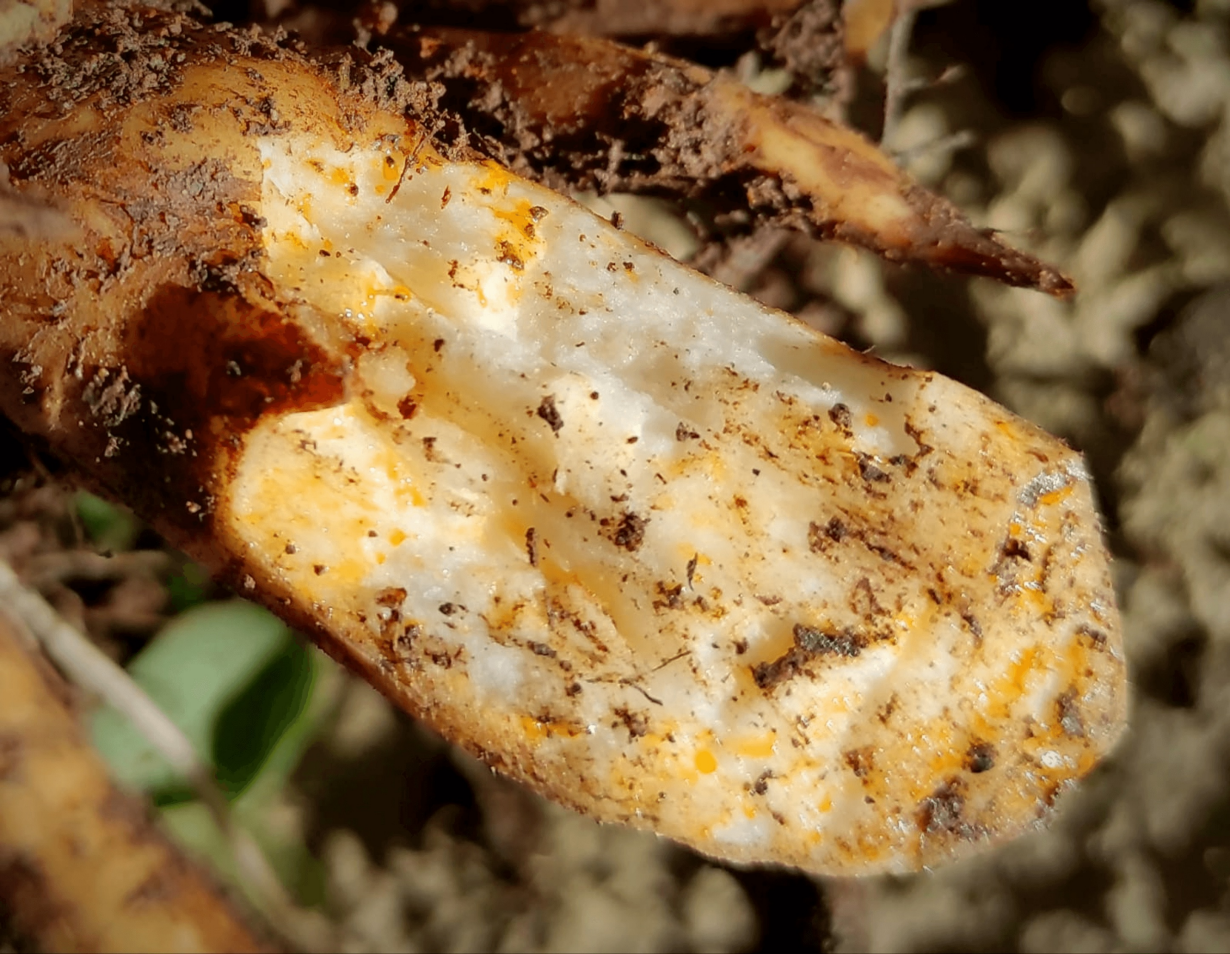
Poison hemlock root detail showing the absence of the characteristic root chambers of water hemlock (Oenanthe crocata).

*C. maculatum* typically grows to a height of about 2 m. The stem is large and hollow with purple spots that are characteristic (Fig. 1), thus one of its usual names, spotted hemlock. The leaves are numerous and tripinnate, and the upper leaves are smaller nearly stalkless, dipinnate, or pinnate. When in inflorescence (from June to September), the umbels are small and have a terminal position, and the petals of the small flowers are white, with an inflexed point. The root (very similar to carrots) is long and pale yellow (Fig. 2). The plant has a bitter taste and a mousy odor. Any part of the plant is toxic, i.e., its leaves, stems, roots, flowers, or seeds. *C. maculatum* resembles water hemlock (*Cicuta virosa*); they can be distinguished by some morphological properties: the first has a single taproot (Fig. 3), purple spotted stems, and a mousy odor, while the former has a branched root system, with a lateral tuber, no spots, and no odor [2]. They are often confused with each other, as proved by a letter to the editor [3]. Poison hemlock can grow anywhere if there is adequate moisture, often found in pastures, alongside streets and streams. It is native to Europe and Asia [4]. This plant has many common names throughout the world, such as hemlok (England), grande cique (France), odort (Sweden), and cicuta (Argentina, Colombia, Chile, and Portugal). In Portugal, it can be encountered from north to south [5].

Biochemistry

The biologic activity (and odor) of the plant originates from 10 simple piperidinic alkaloids found within poison hemlock. The two piperidinic alkaloids that are thought to have the principal toxic effect are ﻿λ-coniceine and coniine. These substances are structurally like nicotine a pyrrolidine alkaloid and stereospecifically bind to cholinergic ligand-gated sodium channels at nicotinic sites [6]. Similar to nicotine, coniine and related alkaloids have an initial excitatory effect, followed by a depressant effect on the central nervous system and clinical characteristics of these intoxications [7,8,9].

The concentration and proportion of the two alkaloids depends on the season, nutrients available, amount of rainfall, and maturation of the plant. During dry sunny seasons, the average fruit size and toxin amount increases [10].

Pathophysiology

Poison hemlock has a volume of distribution of 1 L/kg, a low protein biding of <5%, and no known active metabolites. It has renal and lung excretion after liver metabolization [3]. The physiologic effects are complex and dose dependent. Most toxicity occurs when coniine and related alkaloids enhance the nicotinic action of acetylcholine by depolarizing voltage-gated nicotinic acetylcholine receptors in the central nervous system, at postganglionic autonomic nerves, neuromuscular junctions, and the adrenal medulla. The muscarinic effects of acetylcholine are typically unaffected. Clinical effects are mainly related to the alkaloids’ agonist properties on nicotinic receptors, which are sodium channels, when stimulated result in sodium influx, membrane depolarization, and action potential propagation [11]. The effect is initially stimulatory but shortly followed by depressant, curare-like, antagonist effects [3]. The toxic dose in humans is thought to be 60 mg of coniine, and the fatal dose is 150-300 mg [12].

Clinical presentation

Signs and symptoms following exposure tend to follow a biphasic pattern. Initially, the stimulatory effect of nicotinic stimulation includes nausea and vomiting, excess salivation, diarrhea, and abdominal pain. In the cardiovascular system, hypertension and tachycardia reflect increased autonomic tone and probably constriction of coronary arteries, and pallor from vasoconstriction in the peripheral blood vessels is also common. Early neurologic effects include ataxia, tremor, restlessness, headache, visual and hearing disturbances, dizziness, confusion, muscle fasciculation, miosis, seizures, diaphoresis, and tachypnea. The initial stimulatory effect is classically followed by a more pronounced inhibitory phase caused by the “paradoxical” inhibition of the nicotinic cholinergic receptors. Hypotension, bradycardia, cardiac brady-arrythmias, and ventricular fibrillation can ensue. Central effects are characterized by stupor and coma. Increasing neuromuscular blockage can lead to ptosis, muscular weakness, and paralysis, while bradypnea, apnea, and respiratory failure, alongside with cardiovascular collapse, is the cause of death [13]. Rhabdomyolysis has been reported, and acute kidney injury and renal failure are specific symptoms that are only reported in human poisoning [14]. The onset of symptoms typically begins within 60-90 minutes of ingestion, and in some cases, it may be delayed up to four hours before clinical effects. In mild exposure, symptoms vanish in one to two hours but may persist for 24-72 hours or more in severe intoxications [15,16]. Severe toxicity managed with ventilatory support and supportive care has resulted in complete recovery [3].

Diagnosis

Poisoning from this plant should be a diagnostic consideration in any patient with nicotinic signs and symptoms or ascending paralysis after ingestion of the “plant material." No specific diagnostic test is routinely available. Signs of rhabdomyolysis and transient elevation of liver enzymes are common. The excretion is done by the lungs and kidneys giving the breath and urine the plant characteristic of “mousy” odor. Analysis for coniine can be performed on urinary and blood samples. Diagnostic confirmation is often made by obtaining a portion of the ingested plant [17].

Treatment

The treatment is supportive, and no antidote is available currently. Gastrointestinal lavage and activated charcoal can be used within one hour of ingestion, although without strong evidence that this reduces absorption. The mainstay of treatment of severe intoxication is hemodynamic optimization and mechanical ventilation - likely early in the clinical course [18]. Rhabdomyolysis should be managed leges artis, with aggressive volume replacement and high urine output; hemoperfusion or hemodialysis has been used without clinical or experimental support [18]. As for all intoxications, anti-venom center or institution should be contacted for specialist discussion.

Poison hemlock (*C. maculatum*), from the Apiaceae family, is regarded as one of the most poisonous plants in Europe. Its toxicity is related to nicotinic alkaloids, the most potent being coniine. All parts of the plant are poisonous, and its toxicity is related to the age of the plant, season, and precipitation. During dry sunny seasons, the concentration of toxic alkaloids is the highest [10]. This was probably one of the reasons that the patients presented in this case report did not have more serious intoxication, as ingestion occurred during winter.

As described in an 11-case report in Italy, usually, people are poisoned indirectly by eating small birds or rabbits that have consumed hemlock buds. Intoxication can happen even after the animals were frozen for storage [18].

Poison hemlock should not be confused with water hemlock (*Oenanthe crocata*), which is also a very poisonous plant with GABAergic system depression by its main toxin, the oenanthotoxin [19]. In Portugal, the former - known as Embude - is used as an ancestral technique for fishing in rivers [20], namely, in this southeast part of the country. Popular told the first responding team that the consumed plant was this one. To enhance the importance of recognizing the correct species, in our case, for the first hours of treatment with thought leading to a water hemlock poisoning and only after reaching the photographs of the specific plants and roots (see Fig. 3), we understood to be leading with *C. maculatum*.

As in many other case reports, our two patients had full recovery [21,22,23], even when a fatal dose of toxin is thought to be ingested and mechanical ventilation is needed [24]. Besides the amount of toxin, one of the most important variables for the success of treatment and full recovery is timely and proper medical support. An Australian case report enhances the fact that timely diagnosis is of crucial importance for the outcome [25] and postmortem report of a 27-year-old female found deceased in a relatively isolated area of California [26].

Teratogenicity of piperidine alkaloids is well-studied and documented in neonate animals [27]. In humans, its teratogenic burden is not well-established but believed to be present.

Most of the literature about this toxic plant and its effects on humans arise from clinical cases like ours.

## Conclusions

*C. maculatum* is a very common plant of invasive character and can easily be misidentified as edible species, such as celery, parsnip (for their leaves), or carrots (for their roots). Toxicity comes from piperidine alkaloids, which mimic the nicotine effect on the autonomous nervous system, giving a clinical picture of nicotinic syndrome. Acute intoxication has classically an excitatory phase and a second inhibitory phase whose extreme end is respiratory arrest, which, when found, is the cause of death. Rapid diagnosis and medical support are the mainstay of a successful treatment.
